# Muscle or Heart? Functional Impact of Sarcopenia and Heart Failure in Geriatric Inpatients

**DOI:** 10.3390/jcm14124288

**Published:** 2025-06-16

**Authors:** Valer Donca, Dana Crişan, Camelia Alexandra Coada, Laurenţiu Stoicescu, Elena Buzdugan, Alin Grosu, Mihai Lupu, Lucreţia Avram

**Affiliations:** 1 Geriatrics—Gerontology, Department 5—Medical Specicalties, Faculty of Medicine, Iuliu Haţieganu University of Medicine and Pharmacy, 400012 Cluj-Napoca, Romania; valer.donca@umfcluj.ro (V.D.); avram.lucretia@umfcluj.ro (L.A.); 2 Department of Internal Medicine, 5th Medical Clinic, Faculty of Medicine, Iuliu Haţieganu University of Medicine and Pharmacy, 400012 Cluj-Napoca, Romania; crisan.dana@umfcluj.ro (D.C.); laurentiu.stoicescu@umfcluj.ro (L.S.); cristina.buzdugan@umfcluj.ro (E.B.); grosu.alin@umfcluj.ro (A.G.); 3 Department of Morpho-Functional Sciences, Iuliu Haţieganu University of Medicine and Pharmacy, 400012 Cluj-Napoca, Romania; lupu.mihai@umfcluj.ro

**Keywords:** sarcopenia, heart failure, elderly, geriatric assessment, GerEVal

## Abstract

**Objectives:** To evaluate the individual and combined impact of sarcopenia (SARC) and heart failure (HF) on functional performance, systemic biomarkers, and structural cardiac changes in hospitalized older adults. A total of 598 patients aged ≥65 years admitted to a geriatric unit between January 2023 and December 2024, stratified into four groups based on the presence or absence of sarcopenia and HF. **Methods:** Muscle strength (handgrip), gait speed, SPPB score, and calf circumference were assessed, along with inflammatory/nutritional markers and echocardiographic parameters. Sarcopenia was diagnosed according to EWGSOP2 criteria and HF was diagnosed according to ESC 2021 guidelines. **Results:** Functional impairment was the most severe in the SARC+/HF+ group, with significantly lower handgrip strength, gait speed, and SPPB scores (*p* < 0.001). Sarcopenia alone was associated with greater functional decline than HF alone. Serum hemoglobin and albumin levels were reduced in sarcopenic groups, while NT-proBNP and cardiac remodeling indices (IVS and LVEDD) were highest in patients with both conditions. **Conclusions:** Sarcopenia exerts a significant impact on physical performance in older adults, surpassing that of HF in isolation. The coexistence of sarcopenia and HF amplifies vulnerability and clinical decline, supporting the need for integrated assessment and early muscle-targeted interventions in geriatric cardiology.

## 1. Introduction

Sarcopenia, defined as a progressive and generalized loss of skeletal muscle mass, strength, and function, is increasingly recognized as a major contributor to morbidity and mortality among older adults. While initially viewed as a condition strictly related to aging, recent research has highlighted its systemic relevance, particularly in populations with chronic diseases such as cardiovascular disorders [1,2,3]. This decline in muscle quality and performance not only accelerates physical dependence and frailty, but also interacts with multiple organ systems, including the cardiovascular system. The interdependence of skeletal muscle function and cardiac health has gained increasing attention in recent years. Sarcopenia shares several pathophysiological mechanisms with cardiovascular disease, including chronic inflammation, insulin resistance, endothelial dysfunction, and oxidative stress [4,5]. Moreover, reduced metabolic flexibility and impaired muscular oxygen uptake in individuals with sarcopenia may aggravate clinical manifestations of heart failure (HF), such as exertional dyspnea, fatigue, and reduced functional performance, even in the absence of overt cardiac decompensation [6,7]. Current evidence suggests that the prevalence of sarcopenia among patients with heart failure ranges from 20% to 50%, depending on the diagnostic criteria and population characteristics [8,9]. The prevalence is notably higher among older individuals, particularly those with heart failure with preserved ejection fraction (HFpEF), in whom symptoms such as fatigue and limited exercise tolerance are frequently attributed solely to cardiac dysfunction but may also stem from peripheral skeletal muscle deterioration [10]. Several studies have underscored the prognostic implications of sarcopenia in patients with cardiovascular disease. Sarcopenia has been associated with increased rates of hospital readmission, poor postoperative recovery, reduced survival, and diminished quality of life in both medical and surgical settings [11,12]. Even in patients with preserved ejection fraction, their diminished muscle mass and strength can significantly reduce their physiological reserve, hinder their rehabilitation, and exacerbate their frailty [13].

In recent years, diagnostic strategies for sarcopenia have evolved. The revised EWGSOP2 algorithm recommends prioritizing assessments of muscle strength (e.g., handgrip strength), muscle mass (e.g., appendicular skeletal muscle mass measured via DXA or BIA), and physical performance (e.g., gait speed or SPPB) (1). In cardiogeriatric practice, however, more feasible tools such as the SARC-F questionnaire or biochemical markers like the serum creatinine-to-cystatin C ratio (the Sarcopenia Index, SI) have gained prominence for their practicality and prognostic utility [14,15]. In addition to its direct clinical consequences, sarcopenia has been linked to broader markers of systemic aging and multimorbidity. A large-scale study from the UK Biobank demonstrated that the coexistence of sarcopenia and chronic kidney disease significantly increases the risk of heart failure, stroke, and cardiovascular mortality compared to either condition alone [16]. Notably, sarcopenia may progress silently, even in patients with seemingly normal BMI, due to the fat infiltration of skeletal muscle (myosteatosis), which is commonly observed in sarcopenic obesity [16,17].

Despite growing awareness, sarcopenia remains underdiagnosed and undertreated in cardiovascular care. Its symptoms often overlap with those of heart failure—particularly HFpEF—making it challenging to attribute functional decline solely to muscular or cardiac causes without comprehensive evaluation. Accordingly, there is a growing call to incorporate sarcopenia screening into routine cardiogeriatric assessment, not only to improve diagnostic precision but also to enable early, targeted intervention strategies [18].

This study aimed to investigate the relative contributions of sarcopenia and heart failure to functional decline in older adults, with a particular focus on their combined and independent effects on physical performance, autonomy, and frailty. By analyzing clinical, anthropometric, and functional parameters across stratified patient groups, we sought to determine whether sarcopenia or cardiac dysfunction plays a more dominant role in limiting mobility and daily living capabilities.

## 2. Materials and Methods

### 2.1. Study Design and Patient Inclusion Criteria

This observational study was conducted in the Geriatrics Department of the Municipal Clinical Hospital in Cluj-Napoca, Romania, between 1 January 2023 and 31 December 2024.

Inclusion criteria were as follows: age ≥ 65 years; admission for non-acute, chronic care evaluation within the Geriatrics Department during the defined study period; and completion of a comprehensive standardized geriatric assessment including clinical, functional, laboratory, and body composition evaluations. Eligible patients were required to demonstrate sufficient cognitive and physical ability to participate in standardized strength and mobility testing and to tolerate bioelectrical impedance analysis. Only participants with complete datasets for all prespecified study variables were included.

Participants were excluded if they had acute decompensated illness (including de novo or worsening heart failure), active malignancy, end-stage hepatic or renal disease (eGFR < 30 mL/min/1.73 m^2^), severe cognitive impairment incompatible with standardized testing, or implanted electronic devices contraindicating bioimpedance measurements. All patients provided written informed consent prior to inclusion, and the study was approved by the Ethics Committee of the Municipal Clinical Hospital (approval no. 29/2020). All procedures were conducted in compliance with the ethical principles outlined in the Declaration of Helsinki and its subsequent revisions.

### 2.2. Procedures and Measurements

All patients were evaluated at the time of admission through a standardized global geriatric protocol, which included structured anamnesis, clinical examination, functional and nutritional screening, laboratory testing, and imaging studies.

The diagnosis of sarcopenia was established according to the 2019 revised European Working Group on Sarcopenia in Older People (EWGSOP2) criteria [1] which prioritize muscle strength as the primary parameter. If strength was reduced, muscle quantity and/or physical performance were further assessed to confirm the diagnosis and determine severity.

Muscle strength was measured with a calibrated Baseline^®^ hydraulic hand dynamometer (model MVS-12-0241) in the dominant hand, using standardized positioning and verbal encouragement. Values < 27 kg for men and <16 kg for women indicated low strength. For assessing muscle mass, we used the Visbody-R Explorer device, which performs direct segmental multi-frequency bioelectrical impedance analysis (DSM-BIA) and incorporates 3D posture scanning to improve segmental accuracy. The system provided quantitative estimates of appendicular skeletal muscle mass (ASM), with cut-off values < 7.0 kg/m^2^ for men and <5.5 kg/m^2^ for women. When feasible, physical performance was assessed by usual gait speed over 4 m; values < 0.8 m/s were considered low. In patients unable to complete objective performance tests, the SARC-F questionnaire (score ≥ 4 is suggestive of sarcopenia) was used as an alternative screening tool in line with EWGSOP2 recommendations.

The diagnosis of HF, a clinical syndrome characterized by typical symptoms (e.g., dyspnea and fatigue) and/or signs (e.g., elevated jugular pressure, pulmonary rales, and peripheral edema) resulting from a structural or functional cardiac abnormality, was established according to [19] for the diagnosis and treatment of acute and chronic HF. All echocardiographic evaluations were performed using a Mindray Resona 19 Cardio Cord^®^ (Mindray, Singapore) ultrasound by board-certified cardiologists. In selected cases with inconclusive findings or multiple comorbidities, non-contrast chest computed tomography (CT) was performed to rule out pulmonary causes of dyspnea and support the cardiac diagnosis.

The SARC-F questionnaire was applied as a rapid screening tool for sarcopenia. This five-item instrument assesses self-reported strength, walking assistance, chair rise ability, stair climbing, and fall history. A total score ≥ 4 was considered indicative of probable sarcopenia, in line with the EWGSOP2 recommendations [1].

Handgrip strength (HGS) was measured using a calibrated hydraulic hand dynamometer (Baseline^®^, model MVS-12-0241) applied to the dominant hand, with participants seated in standardized position and verbal encouragement provided. Thresholds of <27 kg for men and <16 kg for women were used to define reduced muscle strength, based on EWGSOP2 diagnostic criteria [1].

Gait speed was evaluated over a 4 m walking distance. A walking speed below 0.8 m/s was classified as low performance and is associated with increased risk of disability, hospitalization, and mortality in older adults [20].

The Short Physical Performance Battery (SPPB) was used to evaluate composite lower-extremity function, including balance, gait speed, and repeated chair rises. The cumulative score ranges from 0 to 12, with lower scores reflecting more severe physical impairment. This tool is well established for predicting mobility limitations and institutionalization risk [21].

The Eastern Cooperative Oncology Group (ECOG) Performance Status Scale was employed to assess overall functional ability. Scores range from 0 (fully active) to 4 (completely disabled), providing a standardized metric for physical dependence and treatment tolerability, particularly relevant in the multimorbid geriatric population [22].

To quantify comorbidity burden, the Cumulative Illness Rating Scale for Geriatrics (CIRS-G) was applied. This instrument rates the severity of impairment across 14 organ systems from 0 (no problem) to 4 (extremely severe), yielding a comprehensive measure of cumulative chronic disease burden [23].

Finally, functional autonomy was assessed using the Activities of Daily Living (ADL) and Instrumental Activities of Daily Living (IADL) scales. The ADL scale evaluates basic self-care abilities such as bathing, dressing, and feeding, while the IADL scale captures more complex tasks including medication management, financial handling, and household activities. These tools are cornerstone measures in geriatric functional assessment, reflecting both physical and cognitive dimensions of independence [24]. Patients were divided based on the presence or absence of sarcopenia and HF, resulting in four groups: no sarcopenia/no HF (SARC−/HF−), sarcopenia/no HF (SARC+/HF−), no sarcopenia/HF (SARC−/HF+), and sarcopenia/HF (SARC+/HF+).

### 2.3. Statistical Analysis

Statistical analyses were performed in R version 4.4.3 (28 February 2025 ucrt)—“Trophy Case” [25]. For continuous variables, descriptive statistics were reported for each variable using median and 25th and 75th percentiles. Normal distribution was tested using the Kolmogorov–Smirnov test, while inequality of variance was tested using Levene’s test. Comparisons between groups were assessed using ANOVA or Kruskal–Wallis tests as appropriate. Post-hoc tests for significant variables were conducted using Tukey or Dunn tests. Categorical variables were reported as absolute frequencies and percentages. Differences between groups were tested using chi-square or Fisher exact tests as appropriate. Standardized residuals were calculated for post-hoc tests. Propensity score matching of the study groups of interest was performed using *MatchIt* R package [26]. A two-sided *p*-value of 0.05 was set to define statistical significance.

## 3. Results

### 3.1. Patients’ Presentation

Out of 1003 patients aged 65 years and older who were admitted and initially evaluated, 598 were included and can be categorized as follows: SARC−/HF− (n = 240), SARC+/HF− (n = 292), SARC−/HF+ (n = 14), and SARC+/HF+ (n = 52) (Table 1). The median BMI was slightly lower in patients with sarcopenia, although not statistically significant across groups (*p* = 0.075). The sex distribution was similar (*p* = 0.235), with females accounting for over 70% of participants in all groups except SARC+/HF+ (57.7%). Patients with sarcopenia (SARC+/HF− and SARC+/HF+) were significantly older than those without (*p* < 0.001). In terms of socioeconomic and living conditions, no significant differences in residence (*p* = 0.270), living arrangement (*p* = 0.748), or type of housing (*p* = 0.090) were seen across groups. However, a slightly higher frequency of patients living on higher floors without elevators was noted in the groups without sarcopenia (SARC−/HF− 21.67% and SARC−/HF+ 28.57%).

Cardiac comorbidity significantly differed across groups (*p* < 0.001), with the highest burden observed in the SARC+/HF+ group, in which >33% were categorized as grade 3 or higher. Hematologic conditions also varied significantly (*p* = 0.048), being more frequent in sarcopenic patients. The prevalence of psychiatric comorbidities also differed significantly (*p* = 0.001), with SARC+/HF+ patients presenting the highest frequency of moderate-to-severe psychiatric disease (up to 29.4% with grade 3) (Table 1 and Table S1). The other comorbidity domains, i.e., vascular, respiratory, gastrointestinal, renal, and neurological, did not differ significantly between groups.

### 3.2. Inflammatory and Nutritional Biomarkers

The analysis of laboratory parameters revealed relevant differences between groups, particularly concerning hematologic and nutritional status. Median hemoglobin values were significantly lower in patients with sarcopenia (SARC+/HF− group (12.5 g/dL (11.1; 13.7)); SARC+/HF+ (12.75 (11.7; 13.8))) compared to non-sarcopenic groups (*p* = 0.001). Although the white blood cell count did not differ significantly (*p* = 0.436), the serum albumin concentration was reduced in groups with both sarcopenia and heart failure, reaching the lowest values in the SARC+/HF+ group (4.0 g/dL [3.7; 4.3]; *p* < 0.001), suggesting an inflammatory or nutritionally deficient profile.

The total cholesterol exhibited a decreasing trend among sarcopenic patients, with lower median values in the SARC+/HF+ group (160 mg/dL (129; 195)) compared to the SARC−/HF− group (173 (150; 203)), though without reaching statistical significance (*p* = 0.150). These findings may reflect underlying metabolic alterations associated with a catabolic state or concomitant comorbidities (Table 2 and Table S1).

### 3.3. Structural and Biomarker Evidence of Cardiac Involvement

Cardiac biomarkers and echocardiographic parameters showed significant differences across groups. Median NT-proBNP levels were markedly elevated in the SARC+/HF+ group (3431 pg/mL (502; 5209)) compared to the other groups, including the SARC−/HF− (381 (115.29; 907)) and SARC+/HF− (675 (244; 2406)) groups (*p* < 0.001), indicating pronounced neurohormonal activation in the context of coexisting conditions. Interventricular septal thickness (IVS) and posterior wall thickness (PWT) were higher in groups with heart failure (*p* = 0.023 and *p* = 0.047, respectively), and the left ventricular end diastolic diameter (LVEDD) reached its highest values in the SARC+/HF+ group (50 mm (46; 53)) (*p* < 0.001), suggesting that cardiac remodeling is associated with a dual diagnosis (Table 3 and Table S1).

### 3.4. Functional Performance Results

ECOG performance status differed significantly between groups (*p* < 0.001). A higher proportion of patients in the SARC+/HF+ group had severe limitations, with approx. 50% having an ECOG performance status ≥ 3. Interestingly, complete functional independence (an ECOG status of 0) was lower in the SARC+/HF− group (6.85%) than in the SARC−/HF+ group (14.29%) (Table 4 and Table S1).

Moreover, functional assessments revealed significant impairments in patients with SARC, HF, or both. Calf circumference was significantly lower in the sarcopenic and/or HF groups (*p* = 0.032). However, when adjusted for BMI, the corrected calf circumference showed no statistically significant difference across groups (*p* = 0.140).

The burden of functional limitation, as estimated by the SARC-F score, was significantly higher among individuals with sarcopenia and/or HF (<0.001), with the highest values observed in SARC+/HF+ patients (median (q1; q3) = 6 (5; 7)). Similarly, the ISHII score was significantly elevated in sarcopenic groups (*p* < 0.001). Handgrip strength, both when estimated and when directly measured using the dominant hand, was significantly reduced in sarcopenic patients. Median right-hand HGS was 22 kg (18; 28) in the SARC−/HF− group compared to 14 kg (10; 18) in the SARC+/HF+ group (*p* < 0.001). The estimated HGS also differed significantly between groups (*p* = 0.016) (Table 4 and Table S1).

Performance-based assessments further confirmed this functional decline. The gait speed (4 m walk test) was significantly slower in sarcopenic and HF patients, particularly in the SARC+/HF+ group (0.34 m/s (0.14; 0.67)). Interestingly, SARC+/HF− patients had a tendency to have lower results (0.4 (0.16; 0.74)) than patients from the SARC−/HF+ group (0.56 (0.34; 0.74)), but without reaching significance in the post-hoc analysis (*p* = 0.292). Similarly, the time to complete five chair rises was increased in the SARC+/HF− group (27 (16.2; 60)) compared to the SARC−/HF+ group (18.42 (16; 23) (*p* = 0.452). Overall, the SPPB scores significantly declined among the groups with the lowest values, especially in SARC+/HF+ patients (*p* < 0.001). Patients with SARC+/HF− had slightly reduced scores when compared to SARC−/HF+ patients (5 (2; 7) vs. 6 (4; 7); *p* = 0.526) (Table 4 and Table S1).

Interestingly, neither the MPI nor SMI differed significantly between groups (*p* = 0.184 and *p* = 0.200, respectively), suggesting that deficits in physical performance may be more prominent than variations in muscle mass alone (Table 4 and Table S1).

We further assessed the correlation between SPPB, TUG test (as sarcopenia parameters), LVEF (as a heart failure parameter), ADL, and FRIED scores. The overall analysis showed a significant impact of all parameters on patient frailty (Table S2). When focusing on each group separately, SPPB scores were significantly associated with ADL scores in both SARC+/HF− (R = −0.773, *p* < 0.001) and SARC−/HF+ (−0.539, *p* = 0.047) patients. Conversely, LVEF showed no significant association, suggesting that cardiac function did not directly influence autonomy in this group.

Similarly, FRIED scores were significantly correlated with SPPB scores and TUG in both SARC+/HF− and SARC−/HF+ patients, further reinforcing the relationship between sarcopenia-related physical deficits and frailty status. Interestingly, LVEF again showed no significant association with patient frailty in SARC+/HF− patients (R = −0.048, *p* = 0.410) but had a significant impact in SARC−/HF+ patients (R = 0.644, *p* = 0.013), suggesting that in patients with heart failure but no sarcopenia, reduced cardiac function also contributes to frailty status, together with muscle functional status (Table S2).

### 3.5. The Independent Impact of Sarcopenia and Heart Failure on Patients’ Performance

Given the important difference in group sizes, we conducted propensity score matching to reduce the imbalance between group sizes and control for confounding variables. Matching (using a 1:3 ratio, without replacement) was based on clinically significant variables among SARC+/HF− and SARC−/HF+ patients, including age, hemoglobin, CK, and vitamin D levels (Table S3, Figure S1). After matching, comparisons between the two groups revealed statistically significant differences in both frailty and autonomy scores. In detail, the FRIED frailty score was significantly higher in the SARC+/HF− group compared to the SARC−/HF+ group (median (q1; q3) = 3 (2; 4) vs. 2 (1; 3), *p* = 0.046). Similarly, the ADL score was higher in the SARC+/HF− group, indicating a greater degree of dependency (median (q1; q3) = 2.5 (0; 11) vs. 0 (0; 0), *p* = 0.032). No significant differences were seen in TUG (18 (13; 22) vs. 19 (15.5; 21.5) *p* = 0.973), SPPB walk test (0.33 (0.14; 0.8) vs. 0.56 (0.34; 0.76) *p* = 0.242), SPPB chair test (27 (17.14; 60) vs. 18.42 (16.75; 32.25) *p* = 0.401) performance (Table S4). Nevertheless, there was a significant difference in SPPB groups, with 29.09% of the SARC+/HF− patients being categorized into the intermediate/high groups with respect to 35.71% of patients from the SARC−/HF+ group (*p* < 0.001).

## 4. Discussion

In this study, we evaluated the interplay between SARC and HF in impacting the physical performance and function of older adults. Our results showed that patients with both SARC and HF (SARC+/HF+) had the most pronounced impairments across all functional domains, including mobility (gait speed), strength (handgrip), endurance (chair stand), and independence in daily living activities (IADLs). These results further support the concept of an additive/synergistic impact of SARC and HF on skeletal muscle dysfunction and functional decline. Moreover, a novel aspect of our analysis lies in the comparative performance of the SARC+/HF− vs. SARC−/HF+ groups, which offers important insights into the differential impact of each condition when present in isolation. Although HF is a well-recognized contributor to exercise intolerance, our data show that sarcopenia alone (SARC+/HF−) is associated with a trend towards an increased functional impairment than isolated HF (SARC−/HF+), especially in lower body strength and endurance, as measured by the chair stand test. The median chair stand time in the SARC+/HF− group was 27 s (q1; q3 16.2; 60), slightly higher than the 18.42 s (q1; q3 16; 23) observed in the SARC−/HF+ group. The relatively better performance of the SARC−/HF+ group compared to the SARC+/HF− group in the physical tests implies that muscle quality deterioration driven by aging and sarcopenia may precede or even outweigh the functional consequences of HF alone, at least in our study population. This suggests that SARC, even in the absence of significant cardiac dysfunction, may lead to a more pronounced effect on muscular function and mobility. Additionally, despite similar muscle mass indices between the two groups, their functional capacity differed, further supporting the concept that muscle mass is not a sufficient indicator for muscle function and emphasizing the need to assess strength and performance directly in clinical evaluations. Moreover, a correlation analysis of SPPB, TUG, and LVEF performance in the SARC−/HF+ group showed a significant association with the FRIED score even in the absence of clinically overt sarcopenia.

From a mechanistic standpoint, our findings reflect the different yet somewhat overlapping pathways of muscle degradation in HF and sarcopenia. In advanced HF, histopathological studies have consistently described a condition known as cardiac skeletal myopathy, characterized by myofiber atrophy, reduced capillary density, and a change from type I oxidative fibers to type II glycolytic fibers, ultimately impairing patients’ aerobic capacity and lowering their exercise tolerance [2,27]. On the other hand, sarcopenia is predominantly an age-related neurogenic disease, in which early denervation and the preferential loss of type II fibers lead to progressive atrophy and decreased contractile function. By 80 years of age, approximately 60% of muscle fibers may be lost, with significant fat and fibrotic infiltration compounding muscle quality deterioration [28]. The convergence of these distinct pathological processes in the SARC+/HF+ group may explain the significant declines observed in the 4 m gait speed, SPPB score, and IADL performance. Importantly, our data show that handgrip strength, an early marker of sarcopenia [29], is further reduced in these patients, suggesting a combined impact of both diseases [30]. This is further supported by the fact that SMI did not differ significantly between groups while functional outcomes were indeed impaired, highlighting the concept that muscle quality and neuromuscular coordination, rather than muscle mass alone, are cumulative determinants of functional capacity [17].

### 4.1. Clinical Implications

In clinical practice, sarcopenia may remain underrecognized, especially when it coexists with HF or other cardiac issues [31,32]. However, our findings emphasize routine screening and early intervention in sarcopenia, even in patients without cardiovascular disease, given its significant impact on function. Another important aspect worth discussing further is the common clinical perception that attributes physical activity intolerance primarily to cardiac dysfunction in geriatric patients. Despite the clear functional limitations observed in our patients with HF, our findings challenge this vision. In everyday practice, clinicians often attribute symptoms such as fatigue or breathlessness to cardiac insufficiency [33]. As a result, patients are not actively encouraged to engage in even low/moderate intensity physical activity or structured exercise programs. However, our data suggest that this approach might be misguided, as sarcopenia could pose a significant limitation to physical function in many of these patients rather than those with HF alone. In fact, as demonstrated by the worse chair stand performance of the SARC+/HF− group compared to the SARC−/HF+ group, sarcopenia alone can impose a greater barrier to mobility than isolated heart failure. Therefore, rather than restricting activity, clinicians should proactively encourage tailored exercise interventions, including resistance and balance training, which have proven benefits in reversing sarcopenia and are also safe and beneficial in the context of stable heart failure [34,35]. A shift from the canonical way of thinking is needed to move from risk-avoidant to proactive rehabilitation strategies, knowing that promoting movement is essential not only for muscle health but also for cardiovascular conditioning and overall quality of life [36,37], especially in this population of patients already prone to generalized muscle loss.

### 4.2. Biochemical and Inflammatory Profile Associated with Sarcopenic and HF Patients

The biochemical alterations observed in our analysis, particularly hypoalbuminemia and low hemoglobin levels in sarcopenic patients, may reflect an underlying state of chronic inflammation and nutritional insufficiency, both commonly encountered in geriatric syndromes. Serum albumin, while traditionally considered a nutritional marker, is increasingly recognized as a negative acute-phase reactant whose levels are influenced by systemic inflammation, hepatic congestion, and comorbidity-related catabolic states. This observation is consistent with reports highlighting the co-occurrence of hypoalbuminemia, anemia, and elevated inflammatory markers (e.g., CRP and IL-6) in advanced sarcopenia or cardiac cachexia [18]. In fact, CRP was also significantly different among groups (*p* < 0.001). Anemia, similarly, may reflect a multifactorial impairment involving reduced erythropoietin production, iron sequestration, and chronic low-grade inflammation, all of which contribute to reduced exercise tolerance and skeletal muscle hypoxia in elderly patients [38].

Of particular interest is the role of vitamin D levels in muscle health, which was significantly lower in patients with sarcopenia in our study. Vitamin D plays a critical role in muscle health and has been increasingly recognized as a modifiable factor in the prevention and management of sarcopenia [39]. Low levels of vitamin D have been consistently associated with reduced physical performance, an increased risk of falls, and accelerated muscle wasting in older adults [39,40]. Given that supplementation has shown beneficial effects in preventing the onset of sarcopenia [40], optimizing vitamin D status in these patients may be a valuable strategy in mitigating sarcopenia and its functional consequences.

### 4.3. Interplay Between Sarcopenia and Cardiac Dysfunction: Biomarkers and Structural Changes

As for cardiac biomarkers, the elevated NT-proBNP levels observed in the SARC+/HF+ group are indicative of increased neurohormonal stress. However, this peptide is known to be nonspecific in older adults, women, and individuals with reduced skeletal muscle mass, potentially confounding its diagnostic interpretation in sarcopenic patients [41]. Moreover, recent evidence suggests that sarcopenia may independently increase natriuretic peptide levels through mechanisms related to altered muscle metabolism, fluid redistribution, and impaired renal clearance. Accordingly, NT-proBNP values in sarcopenic heart failure patients may reflect not only hemodynamic overload but also systemic catabolic and inflammatory stress [42].

The echocardiographic findings in our study reinforce the association between skeletal muscle decline and adverse cardiac remodeling. Increased IVS, PWT, and LVEDD in SARC+/HF+ patients suggest that sarcopenia may potentiate concentric hypertrophy and diastolic dysfunction. These changes are consistent with prior studies describing enhanced RAAS activation, oxidative stress, and myostatin overexpression as shared pathways contributing to both sarcopenia and cardiac structural alterations [18]. This overlap between muscular and cardiac pathophysiology complicates both differential diagnosis and therapeutic decisions. Thus, reliance on biomarkers such as NT-proBNP or serum albumin alone is insufficient; a comprehensive assessment incorporating functional performance, body composition, and targeted imaging is required to accurately stratify risk in geriatric patients with overlapping syndromes [43].

### 4.4. Study Limitations and Future Perspectives

The main limitation of our study stems from its observational nature, as it limits our ability to infer causality between sarcopenia, heart failure, and impairments in physical performance or functional status. Next, the study was conducted at a single center, which may limit the generalizability of the findings to broader populations. Moreover, the subgroups of patients with HF had small sample sizes, especially the group of patients without sarcopenia but with HF (SARC−/HF+ n = 14). This represents the most important limitation of our study, as the small numbers may have reduced the statistical power to detect meaningful differences between groups. It is also worth noting that even though multiple comorbidities were considered using CIRS-G, residual confounding variables or unmeasured variables (such as medication use, socioeconomic status, or nutritional status) may have influenced the results. Nevertheless, the significant results obtained after the propensity matching of the patients confirmed our initial results. Furthermore, the study did not include circulating biomarkers commonly implicated in sarcopenia and cardiac cachexia, such as TNF-α, IL-6, or myostatin. These markers are known to mediate chronic inflammation, muscle wasting, and anabolic resistance, and their evaluation could have enhanced the pathophysiological interpretation of the observed clinical findings.

While these results are compelling and interesting, they must be interpreted in light of the abovementioned limitations. Longitudinal studies with larger cohorts are required to determine temporal relationships and the strength of their impact on the progression of disability, as well as to identify the best prevention strategies for this class of patients.

## 5. Conclusions

Our study highlights sarcopenia as an important contributing factor to functional impairment in older adults, often associated with a greater impact on physical performance than HF alone. Patients with sarcopenia, even in the absence of HF, scored lower on mobility and independence tests compared to those with HF without sarcopenia. This suggests that skeletal muscle degeneration is an important contributing factor for reduced exercise tolerance in geriatric patients. These findings support the need to reduce the level of sarcopenia in all types of frail patients, with an added beneficial effect in patients with HF. More data are needed to ascertain the direct correlation between sarcopenia treatment and patient outcomes.

## Figures and Tables

**Table 1:** Demographic characteristics of the patients and detailed distribution of comorbidities by CIRS-G domains across study groups.

Variable		SARC−/HF− n = 240	SARC+/HF− n = 292	SARC−/HF+ n = 14	SARC+/HF+ n = 52	*p*-Value
**Demographic characteristics**						
**Age (years), median (q1; q3)**		76.84 (71.01; 81.84)	82.17 (76.91; 86.15)	77.84 (72.97; 84.16)	84.07 (79.03; 88.5)	<0.001
**Sex, n (%)**	**F**	173 (72.08)	205 (70.21)	10 (71.43)	30 (57.69)	0.235
	**M**	67 (27.92)	87 (29.79)	4 (28.57)	22 (42.31)	
**BMI, median (q1; q3)**		29.2 (24.86; 32.72)	27.06 (23.05; 32.09)	28.86 (25.01; 29.76)	27.83 (22.97; 31.22)	0.075
**BMI group, n (%)**	**underweight**	5 (2.1)	8 (2.78)	0 (0)	3 (6.25)	0.673
	**normal**	59 (24.79)	89 (30.9)	3 (21.43)	13 (27.08)	
	**overweight**	74 (31.09)	84 (29.17)	7 (50)	15 (31.25)	
	**obese**	82 (34.45)	91 (31.6)	4 (28.57)	16 (33.33)	
	**morbid**	18 (7.56)	16 (5.56)	0 (0)	1 (2.08)	
**Residence, n (%)**	**Rural**	89 (37.08)	120 (41.1)	4 (28.57)	26 (50)	0.270
	**Urban**	151 (62.92)	172 (58.9)	10 (71.43)	26 (50)	
**Housing, n (%)**	**House**	139 (57.92)	186 (63.7)	6 (42.86)	34 (65.38)	0.090
	**Flat, ground floor**	12 (5)	13 (4.45)	3 (21.43)	1 (1.92)	
	**Higher floor, with elevator**	36 (15)	31 (10.62)	1 (7.14)	8 (15.38)	
	**Higher floor, no elevator**	52 (21.67)	53 (18.15)	4 (28.57)	8 (15.38)	
	**Institutionalized**	1 (0.42)	9 (3.08)	0 (0)	1 (1.92)	
**Lives alone, n (%)**	**Alone**	6 (2.5)	5 (1.71)	0 (0)	0 (0)	0.748
	**Alone with support**	98 (40.83)	106 (36.3)	7 (50)	20 (38.46)	
	**With others**	136 (56.67)	181 (61.99)	7 (50)	32 (61.54)	
**Smoking status, n (%)**	**Never**	175 (72.92)	225 (77.32)	12 (85.71)	38 (73.08)	0.262
	**Past**	45 (18.75)	55 (18.9)	2 (14.29)	13 (25)	
	**Current**	20 (8.33)	11 (3.78)	0 (0)	1 (1.92)	
**Comorbidity burden**						
**CIRS G Heart, n (%)**	**0**	76 (31.67)	63 (21.72)	0 (0)	4 (7.69)	<0.001
	**1**	8 (3.33)	12 (4.14)	2 (14.29)	2 (3.85)	
	**2**	134 (55.83)	179 (61.72)	8 (57.14)	28 (53.85)	
	**3**	20 (8.33)	30 (10.34)	3 (21.43)	15 (28.85)	
	**4**	2 (0.83)	6 (2.07)	1 (7.14)	3 (5.77)	
**CIRS G Vascular, n (%)**	**0**	13 (5.42)	16 (5.5)	1 (7.14)	3 (5.77)	0.912
	**1**	25 (10.42)	22 (7.56)	0 (0)	2 (3.85)	
	**2**	46 (19.17)	59 (20.27)	3 (21.43)	9 (17.31)	
	**3**	156 (65)	193 (66.32)	10 (71.43)	38 (73.08)	
	**4**	0 (0)	1 (0.34)	0 (0)	0 (0)	
**CIRS G Hematopoietic, n (%)**	**0**	169 (70.42)	165 (56.7)	11 (78.57)	30 (57.69)	0.048
	**1**	48 (20)	80 (27.49)	1 (7.14)	13 (25)	
	**2**	17 (7.08)	34 (11.68)	1 (7.14)	6 (11.54)	
	**3**	6 (2.5)	11 (3.78)	1 (7.14)	2 (3.85)	
	**4**	0 (0)	1 (0.34)	0 (0)	1 (1.92)	
**CIRS G Respiratory, n (%)**	**0**	183 (76.25)	218 (74.91)	14 (100)	35 (67.31)	0.096
	**1**	22 (9.17)	17 (5.84)	0 (0)	1 (1.92)	
	**2**	23 (9.58)	33 (11.34)	0 (0)	10 (19.23)	
	**3**	2 (0.83)	4 (1.37)	0 (0)	3 (5.77)	
	**4**	10 (4.17)	19 (6.53)	0 (0)	3 (5.77)	
**CIRS G Ears, nose, throat, and larynx, n (%)**	**0**	64 (26.67)	40 (13.75)	1 (7.14)	7 (13.46)	<0.001
	**1**	134 (55.83)	144 (49.48)	7 (50)	28 (53.85)	
	**2**	36 (15)	91 (31.27)	5 (35.71)	14 (26.92)	
	**3**	5 (2.08)	15 (5.15)	1 (7.14)	3 (5.77)	
	**4**	1 (0.42)	1 (0.34)	0 (0)	0 (0)	
**CIRS G upper GI, n (%)**	**0**	147 (61.25)	187 (64.26)	9 (64.29)	32 (61.54)	0.991
	**1**	60 (25)	63 (21.65)	4 (28.57)	13 (25)	
	**2**	24 (10)	30 (10.31)	1 (7.14)	4 (7.69)	
	**3**	6 (2.5)	6 (2.06)	0 (0)	2 (3.85)	
	**4**	3 (1.25)	5 (1.72)	0 (0)	1 (1.92)	
**CIRS G lower GI, n (%)**	**0**	132 (55)	138 (47.42)	7 (50)	23 (44.23)	0.356
	**1**	93 (38.75)	123 (42.27)	6 (42.86)	27 (51.92)	
	**2**	10 (4.17)	18 (6.19)	0 (0)	2 (3.85)	
	**4**	5 (2.08)	12 (4.12)	1 (7.14)	0 (0)	
**CIRS G Liver, pancreas, and biliary, n (%)**	**0**	216 (90)	262 (90.03)	14 (100)	44 (84.62)	0.824
	**1**	8 (3.33)	13 (4.47)	0 (0)	4 (7.69)	
	**2**	14 (5.83)	13 (4.47)	0 (0)	4 (7.69)	
	**3**	2 (0.83)	3 (1.03)	0 (0)	0 (0)	
**CIRS G Renal, n (%)**	**0**	202 (84.17)	235 (80.76)	11 (78.57)	37 (71.15)	0.161
	**1**	15 (6.25)	14 (4.81)	0 (0)	4 (7.69)	
	**2**	11 (4.58)	19 (6.53)	0 (0)	4 (7.69)	
	**3**	10 (4.17)	20 (6.87)	2 (14.29)	7 (13.46)	
	**4**	2 (0.83)	3 (1.03)	1 (7.14)	0 (0)	
**CIRS G Genitourinary, n (%)**	**0**	95 (39.58)	97 (33.45)	5 (35.71)	17 (32.69)	0.085
	**1**	88 (36.67)	108 (37.24)	6 (42.86)	17 (32.69)	
	**2**	48 (20)	75 (25.86)	1 (7.14)	15 (28.85)	
	**3**	2 (0.83)	1 (0.34)	2 (14.29)	1 (1.92)	
	**4**	7 (2.92)	9 (3.1)	0 (0)	2 (3.85)	
**CIRS G Musculoskeletal and skin, n (%)**	**0**	159 (66.25)	195 (67.24)	9 (64.29)	31 (59.62)	0.641
	**1**	40 (16.67)	40 (13.79)	3 (21.43)	7 (13.46)	
	**2**	13 (5.42)	26 (8.97)	1 (7.14)	6 (11.54)	
	**3**	23 (9.58)	19 (6.55)	1 (7.14)	6 (11.54)	
	**4**	5 (2.08)	10 (3.45)	0 (0)	2 (3.85)	
**CIRS G Neurologic, n (%)**	**0**	141 (58.75)	172 (59.31)	8 (57.14)	32 (61.54)	0.990
	**1**	28 (11.67)	32 (11.03)	1 (7.14)	3 (5.77)	
	**2**	61 (25.42)	76 (26.21)	5 (35.71)	15 (28.85)	
	**3**	3 (1.25)	3 (1.03)	0 (0)	0 (0)	
	**4**	7 (2.92)	7 (2.41)	0 (0)	2 (3.85)	
**CIRS G Endocrine and breast, n (%)**	**0**	141 (58.75)	172 (59.31)	8 (57.14)	32 (61.54)	0.993
	**1**	28 (11.67)	32 (11.03)	1 (7.14)	3 (5.77)	
	**2**	61 (25.42)	76 (26.21)	5 (35.71)	15 (28.85)	
	**3**	3 (1.25)	3 (1.03)	0 (0)	0 (0)	
	**4**	7 (2.92)	7 (2.41)	0 (0)	2 (3.85)	
**CIRS G Psychiatric illness, n (%)**	**0**	126 (52.72)	113 (39.37)	7 (50)	19 (37.25)	0.001
	**1**	48 (20.08)	58 (20.21)	5 (35.71)	9 (17.65)	
	**2**	44 (18.41)	69 (24.04)	1 (7.14)	8 (15.69)	
	**3**	16 (6.69)	35 (12.2)	1 (7.14)	15 (29.41)	
	**4**	5 (2.09)	12 (4.18)	0 (0)	0 (0)	

Abbreviations. SARC: sarcopenia; HF: heart failure; n: number of patients; BMI: body mass index; CIRS-G: Cumulative Illness Rating Scale—Geriatric version; GI: gastrointestinal.

**Table 2:** Inflammatory and nutritional laboratory parameters across sarcopenia and heart failure categories.

Variable	SARC−/HF− n = 240	SARC+/HF− n = 292	SARC−/HF+ n = 14	SARC+/HF+ n = 52	*p*-Value
**Blood tests at presentation**					
**Hemoglobin (g/dL), median (q1; q3)**	13.1 (11.9; 14.1)	12.5 (11.1; 13.7)	13.65 (12.7; 14.8)	12.75 (11.7; 13.8)	0.001
**WBC count (×10^3^;/µL), median (q1; q3)**	6.9 (5.83; 8.12)	7.15 (5.7; 8.65)	7.29 (5.98; 7.7)	7.22 (6.01; 8.98)	0.436
**Total cholesterol (mg/dL), median (q1; q3)**	173 (150; 203)	169.5 (140; 202)	161 (127; 184)	160 (129; 195)	0.150
**Triglycerides (mg/dL), median (q1; q3)**	102 (78; 133)	104 (71; 140)	86.5 (76; 98)	93 (76; 120)	0.672
**HDL cholesterol (mg/dL), median (q1; q3)**	48 (39; 56)	45 (37; 55)	47 (40; 53)	45 (35; 52)	0.186
**LDL cholesterol (mg/dL), median (q1; q3)**	110 (87; 139)	104.5 (80; 140)	102 (74; 127)	98 (73; 120)	0.262
**Blood glucose (mg/dL), median (q1; q3)**	105 (95; 118)	104 (94; 122)	108 (97; 153)	104.5 (90; 125)	0.849
**HbA1c (%), median (q1; q3)**	6.04 (5.7; 6.8)	6.1 (5.7; 6.5)	7.35 (6.08; 7.9)	6.8 (6; 8.7)	0.009
**Serum albumin (g/dL), median (q1; q3)**	4.4 (4.1; 4.6)	4.2 (3.9; 4.4)	4.25 (4; 4.6)	4 (3.7; 4.3)	<0.001
**Blood urea (mg/dL), median (q1; q3)**	45 (36; 58)	49 (41; 62)	45 (41; 49)	51 (39; 71)	0.002
**Serum uric acid (mg/dL), median (q1; q3)**	6 (4.8; 7.1)	6 (4.6; 7.5)	5.4 (3.9; 6.9)	6.9 (5.9; 8.3)	0.018
**Serum creatinine (mg/dL), median (q1; q3)**	0.89 (0.73; 1.09)	0.94 (0.74; 1.21)	0.89 (0.76; 0.98)	1 (0.76; 1.19)	0.224
**CK (U/L), median (q1; q3)**	78 (51; 117)	64 (40; 101)	50 (38; 68)	57 (29; 94)	<0.001
**Na+ (mml/L), median (q1; q3)**	140 (138; 141)	140 (138; 141)	140.5 (139; 141)	139 (137; 141)	0.405
**K+ (mmol/L), median (q1; q3)**	4.3 (4; 4.6)	4.4 (4; 4.7)	4.3 (4.1; 4.4)	4.45 (4.1; 4.9)	0.195
**Serum Vitamin D (ng/mL), median (q1; q3)**	23.7 (15.3; 33.51)	20.37 (12.55; 30.5)	27.56 (22.7; 33.65)	14.14 (10.06; 23.02)	<0.001
**CRP (mg/dl), median (q1; q3)**	0.36 (0.17; 0.73)	0.53 (0.18; 2.19)	0.44 (0.36; 1.02)	1.01 (0.42; 3.04)	<0.001

Abbreviations. SARC: sarcopenia; HF: heart failure; n: number of patients; WBC: White blood cells; HDL: High-density lipoprotein; HbA1c: Glycated hemoglobin; CK: Creatine kinase; Na+: Serum sodium; K+: Serum potassium: LDL: Low-density lipoprotein; CRP: C-reactive protein.

**Table 3:** Cardiac biomarkers and echocardiographic parameters in relation to sarcopenia and heart failure.

Variable	SARC−/HF− n = 240	SARC+/HF− n = 292	SARC−/HF+ n = 14	SARC+/HF+ n = 52	*p*-Value
**NTproBNP (pg/mL), median (q1; q3)**	381 (115.29; 907)	675 (244; 2406)	1741 (72.9; 1904)	3431 (502; 5209)	<0.001
**IVS (mm), median (q1; q3)**	12 (11; 13)	12 (12; 13)	13 (12; 14)	12 (11; 13)	0.023
**PWT (mm), median (q1; q3)**	12 (11; 13)	12 (11; 13)	13 (12; 13)	12 (11; 13)	0.047
**LVEDD (mm), median (q1; q3)**	46 (42; 50)	46 (42; 49)	50 (45; 55)	50 (46; 53)	<0.001
**LVESD (mm), median (q1; q3)**	32 (28; 35)	30 (27; 34)	36 (32; 37)	36 (31; 39)	<0.001
**LVEF (%), median (q1; q3)**	55 (50; 60)	55 (50; 59)	40 (37; 40)	40 (35; 42)	<0.001

Abbreviations. SARC: sarcopenia; HF: heart failure; n: number of patients; NT-proBNP: N-terminal pro-B-type natriuretic peptide; IVS: interventricular septal thickness; PWT: posterior wall thickness; LVEDD: left ventricular end diastolic diameter; LVESD: left ventricular end systolic diameter; LVEF: left ventricular ejection fraction.

**Table 4:** Sarcopenia-related functional and morphological parameters, and functional status and instrumental activities of daily living in study participants.

Variable			SARC−/HF− n = 240	SARC+/HF− n = 292	SARC−/HF+ n = 14	SARC+/HF+ n = 52	*p*-Value
**Sarcopenia-related measurements**							
**Calf circumference (cm), median (q1; q3)**			36 (32; 39)	34 (31; 37.5)	35 (30.5; 37)	34 (30.5; 38.5)	0.032
**Corrected calf circumference (cm), median (q1; q3)**			31.35 (29; 34)	31 (28.5; 33)	31 (28; 34.7)	30 (28; 33)	0.140
**SARC-F score, median (q1; q3)**			3 (1; 5)	5 (3; 7)	4 (3; 5)	6 (5; 7)	<0.001
**ISHII, median (q1; q3)**			180.46 (128.53; 210.88)	224 (167.69; 242.19)	184.93 (143.56; 195.09)	214.45 (163.55; 242.61)	<0.001
**HGS estimated (kg), median (q1; q3)**			25.52 (23.53; 38.72)	24.39 (22.41; 37.94)	26.75 (24.03; 27.71)	24.73 (22.03; 38.32)	0.016
**HGS right hand (kg), median (q1; q3)**			22 (18; 28)	15 (10; 18)	19 (18; 21)	14 (10; 18)	<0.001
**MPI, median (q1; q3)**			4.69 (3.88; 5.43)	5.19 (4.1; 6.16)	4.69 (3.66; 4.88)	4.59 (3.55; 5.99)	0.184
**SMI, median (q1; q3)**			45.06 (41.02; 50.19)	45.35 (39.93; 50.82)	44.14 (43.18; 44.66)	39.49 (35.48; 45.22)	0.200
**SPPB 4 m walk speed (sec), median (q1; q3)**			0.73 (0.4; 1)	0.4 (0.16; 0.74)	0.56 (0.34; 0.74)	0.34 (0.14; 0.67)	<0.001
**SPPB chair test (seconds), median (q1; q3)**			17 (13; 24.87)	27 (16.2; 60)	18.42 (16; 23)	45 (18; 60)	<0.001
**SPPB score, median (q1; q3)**			8 (5; 10)	5 (2; 7)	6 (4; 7)	3 (1; 6)	<0.001
**TUG (seconds), median (q1; q3)**			14 (11; 20)	20 (14; 25)	19 (15.5; 21.5)	20 (14.75; 25)	<0.001
**Functional measurements**							
**ECOG, n (%)**	**0. Fully active**		38 (15.83)	20 (6.85)	2 (14.29)	3 (5.77)	<0.001
	**1. Active with mild restrictions**		128 (53.33)	120 (41.1)	6 (42.86)	17 (32.69)	
	**2. Ambulatory**		40 (16.67)	49 (16.78)	2 (14.29)	6 (11.54)	
	**3. Self-care**		33 (13.75)	80 (27.4)	4 (28.57)	19 (36.54)	
	**4. Disabled**		1 (0.42)	23 (7.88)	0 (0)	7 (13.46)	
**FRIED score, median (q1; q3)**			2 (1; 3)	3 (2; 4)	2 (1; 3)	3 (3; 4)	<0.001
**VIVIFRAIL, n (%)**		**A**	35 (15.22)	96 (34.41)	1 (8.33)	24 (48)	<0.001
		**B**	64 (27.83)	92 (32.97)	8 (66.67)	16 (32)	
		**C**	75 (32.61)	63 (22.58)	2 (16.67)	7 (14)	
		**D**	56 (24.35)	28 (10.04)	1 (8.33)	3 (6)	
**ADL score, median (q1; q3)**			0(0;4)	4 (0; 13)	0 (0; 0)	9 (3; 17)	<0.001
***IADL domain*, n (%) **							
**Shopping**		**no**	104 (43.51)	65 (22.26)	3 (21.43)	6 (11.54)	<0.001
		**yes**	135 (56.49)	227 (77.74)	11 (78.57)	46 (88.46)	
**Meal Preparation**		**no**	164 (68.62)	132 (45.21)	7 (50)	14 (26.92)	<0.001
		**yes**	75 (31.38)	160 (54.79)	7 (50)	38 (73.08)	
**Housekeeping**		**no**	133 (55.65)	81 (27.74)	5 (35.71)	10 (19.23)	<0.001
		**yes**	106 (44.35)	211 (72.26)	9 (64.29)	42 (80.77)	
**Laundry**		**no**	169 (70.71)	117 (40.07)	9 (64.29)	14 (26.92)	<0.001
		**yes**	70 (29.29)	175 (59.93)	5 (35.71)	38 (73.08)	
**Mobility**		**no**	135 (56.49)	112 (38.36)	6 (42.86)	11 (21.15)	<0.001
		**yes**	104 (43.51)	180 (61.64)	8 (57.14)	41 (78.85)	
**Medication management**		**no**	196 (82.01)	189 (64.73)	10 (71.43)	29 (55.77)	<0.001
		**yes**	43 (17.99)	103 (35.27)	4 (28.57)	23 (44.23)	
**Financial management**		**no**	166 (69.46)	161 (55.14)	8 (57.14)	18 (34.62)	<0.001
		**yes**	73 (30.54)	131 (44.86)	6 (42.86)	34 (65.38)	

Abbreviations: SARC: sarcopenia; HF: heart failure; n: number of patients; q: quartile; ECOG: Eastern Cooperative Oncology Group Performance Status; SARC-F: Strength, Assistance with walking, Rise from a chair, Climb stairs, and Falls; HGS: handgrip strength; MPI: Multidimensional Prognostic Index; SMI: Skeletal Muscle Index; SPPB: Short Physical Performance Battery; TUG: Timed Up and Go FRIED: Fried frailty criteria; VIVIFRAIL: functional exercise program for older adults; ADL: activities of daily living; IADL: instrumental activities of daily living

## Data Availability

The dataset used during the current study is available from the corresponding author upon reasonable request.

## Supplementary Materials

The following supporting information can be downloaded at: https://www.mdpi.com/article/10.3390/jcm14124288/s1, Figure S1: Presentation of the propensity matching between patients from the SARC+/HF− group and those from the SARC−/HF+ group. A total of 56 SARC+/HF− patients were msthced with the 14 SARC−/HF+ cases; Table S1: Post-hoc analyses of all significant variables; Table S2: Correlation between sarcopenia parameters and LVEF and ADL and FRIED scores among the patients’ groups; Table S3: Summary of the balance assessment; Table S4: Characteristics of the propensity matched patients.
