# Supplementary material for: Muscle or Heart? Functional Impact of Sarcopenia and Heart Failure in Geriatric Inpatients
Source: J Clin Med. 2025 Jun 16;14(12):4288. doi: 10.3390/jcm14124288 (PMC12194708; doi:10.3390/jcm14124288)
Supplement: Supplementary file 1 [file jcm-14-04288-s001.zip › jcm-3681282-supplementary.pdf]

Journal of Clinical Medicine

MDPI

Supplementary Table 1. Post-hoc analyses of all significant variables.

	Compared groups		
**Variable**	**Group 1**	**Group 2**	**p-value**
VIVIFRAIL_reduced (n%)	SARC-/HF-	SARC+/HF-	**<0.001**
	SARC-/HF-	SARC-/HF+	0.073
	SARC-/HF-	SARC+/HF+	**<0.001**
	SARC+/HF-	SARC-/HF+	0.096
	SARC+/HF-	SARC+/HF+	0.260
	SARC-/HF+	SARC+/HF+	**0.042**
IADL_Shopping (n%)	SARC-/HF-	SARC+/HF-	**<0.001**
	SARC-/HF-	SARC-/HF+	0.163
	SARC-/HF-	SARC+/HF+	**<0.001**
	SARC+/HF-	SARC-/HF+	1.000
	SARC+/HF-	SARC+/HF+	0.094
	SARC-/HF+	SARC+/HF+	0.386
IADL_Meal Preparation (n%)	SARC-/HF-	SARC+/HF-	**<0.001**
	SARC-/HF-	SARC-/HF+	0.154
	SARC-/HF-	SARC+/HF+	**<0.001**
	SARC+/HF-	SARC-/HF+	0.788
	SARC+/HF-	SARC+/HF+	**0.015**
	SARC-/HF+	SARC+/HF+	0.117
IADL_Housekeeping (n%)	SARC-/HF-	SARC+/HF-	**<0.001**
	SARC-/HF-	SARC-/HF+	0.173
	SARC-/HF-	SARC+/HF+	**<0.001**
	SARC+/HF-	SARC-/HF+	0.547
	SARC+/HF-	SARC+/HF+	0.234
	SARC-/HF+	SARC+/HF+	0.279
IADL_Laundry (n%)	SARC-/HF-	SARC+/HF-	**<0.001**
	SARC-/HF-	SARC-/HF+	0.564
	SARC-/HF-	SARC+/HF+	**<0.001**
	SARC+/HF-	SARC-/HF+	0.095
	SARC+/HF-	SARC+/HF+	0.088
	SARC-/HF+	SARC+/HF+	**0.013**
IADL_Mobility (n%)	SARC-/HF-	SARC+/HF-	**<0.001**
	SARC-/HF-	SARC-/HF+	0.409
	SARC-/HF-	SARC+/HF+	**<0.001**
	SARC+/HF-	SARC-/HF+	0.782
	SARC+/HF-	SARC+/HF+	**0.018**
	SARC-/HF+	SARC+/HF+	0.165
IADL_Medication management (n%)	SARC-/HF-	SARC+/HF-	**<0.001**
	SARC-/HF-	SARC-/HF+	0.302
	SARC-/HF-	SARC+/HF+	**<0.001**
	SARC+/HF-	SARC-/HF+	0.777
	SARC+/HF-	SARC+/HF+	0.274

	SARC-/HF+	SARC+/HF+	0.367
IADL_Financial Management (n%)	SARC-/HF-	SARC+/HF-	**0.001**
	SARC-/HF-	SARC-/HF+	0.377
	SARC-/HF-	SARC+/HF+	**<0.001**
	SARC+/HF-	SARC-/HF+	1.000
	SARC+/HF-	SARC+/HF+	**0.007**
	SARC-/HF+	SARC+/HF+	0.139
CIRS G Heart (n%)	SARC-/HF-	SARC+/HF-	0.101
	SARC-/HF-	SARC-/HF+	**0.002**
	SARC-/HF-	SARC+/HF+	**<0.001**
	SARC+/HF-	SARC-/HF+	**0.021**
	SARC+/HF-	SARC+/HF+	**0.001**
	SARC-/HF+	SARC+/HF+	0.525
CIRS G Hematopoietic (n%)	SARC-/HF-	SARC+/HF-	**0.015**
	SARC-/HF-	SARC-/HF+	0.325
	SARC-/HF-	SARC+/HF+	0.116
	SARC+/HF-	SARC-/HF+	0.233
	SARC+/HF-	SARC+/HF+	0.664
	SARC-/HF+	SARC+/HF+	0.513
CIRS G Ears, nose, throat and larynx (n%)	SARC-/HF-	SARC+/HF-	**<0.001**
	SARC-/HF-	SARC-/HF+	0.082
	SARC-/HF-	SARC+/HF+	**0.041**
	SARC+/HF-	SARC-/HF+	0.887
	SARC+/HF-	SARC+/HF+	0.922
	SARC-/HF+	SARC+/HF+	0.859
CIRS G Psychiatric illness (n%)	SARC-/HF-	SARC+/HF-	**0.012**
	SARC-/HF-	SARC-/HF+	0.586
	SARC-/HF-	SARC+/HF+	**0.001**
	SARC+/HF-	SARC-/HF+	0.416
	SARC+/HF-	SARC+/HF+	**0.027**
	SARC-/HF+	SARC+/HF+	0.180
CRP (mg/dl)	SARC-/HF-	SARC+/HF-	0.005
	SARC-/HF-	SARC-/HF+	0.998
	SARC-/HF-	SARC+/HF+	0.794
	SARC+/HF-	SARC-/HF+	0.606
	SARC+/HF-	SARC+/HF+	0.816
	SARC-/HF+	SARC+/HF+	0.915
Age (years)	SARC-/HF-	SARC+/HF-	**<0.001**
	SARC-/HF-	SARC-/HF+	0.805
	SARC-/HF-	SARC+/HF+	**<0.001**
	SARC+/HF-	SARC-/HF+	0.355
	SARC+/HF-	SARC+/HF+	0.484
	SARC-/HF+	SARC+/HF+	0.123
Calf circumference (cm)	SARC-/HF-	SARC+/HF-	0.723
	SARC-/HF-	SARC-/HF+	0.969
	SARC-/HF-	SARC+/HF+	0.906
	SARC+/HF-	SARC-/HF+	0.999

	SARC+/HF-	SARC+/HF+	1.000
	SARC-/HF+	SARC+/HF+	1.000
Corrected calf circumference (cm)	SARC-/HF-	SARC+/HF-	0.999
	SARC-/HF-	SARC-/HF+	1.000
	SARC-/HF-	SARC+/HF+	0.995
	SARC+/HF-	SARC-/HF+	1.000
	SARC+/HF-	SARC+/HF+	0.987
	SARC-/HF+	SARC+/HF+	0.997
SARC- F score	SARC-/HF-	SARC+/HF-	**<0.001**
	SARC-/HF-	SARC-/HF+	0.519
	SARC-/HF-	SARC+/HF+	**<0.001**
	SARC+/HF-	SARC-/HF+	0.827
	SARC+/HF-	SARC+/HF+	0.203
	SARC-/HF+	SARC+/HF+	0.282
ISHII score	SARC-/HF-	SARC+/HF-	**<0.001**
	SARC-/HF-	SARC-/HF+	1.000
	SARC-/HF-	SARC+/HF+	0.002
	SARC+/HF-	SARC-/HF+	0.096
	SARC+/HF-	SARC+/HF+	0.970
	SARC-/HF+	SARC+/HF+	0.242
HGS estimated (kg)	SARC-/HF-	SARC+/HF-	0.653
	SARC-/HF-	SARC-/HF+	0.999
	SARC-/HF-	SARC+/HF+	0.995
	SARC+/HF-	SARC-/HF+	0.996
	SARC+/HF-	SARC+/HF+	0.805
	SARC-/HF+	SARC+/HF+	0.994
SPPB walk speed 4m (seconds)	SARC-/HF-	SARC+/HF-	**<0.001**
	SARC-/HF-	SARC-/HF+	0.444
	SARC-/HF-	SARC+/HF+	**<0.001**
	SARC+/HF-	SARC-/HF+	0.811
	SARC+/HF-	SARC+/HF+	0.841
	SARC-/HF+	SARC+/HF+	0.613
SPPB chair test (seconds)	SARC-/HF-	SARC+/HF-	**<0.001**
	SARC-/HF-	SARC-/HF+	0.836
	SARC-/HF-	SARC+/HF+	**<0.001**
	SARC+/HF-	SARC-/HF+	0.647
	SARC+/HF-	SARC+/HF+	0.480
	SARC-/HF+	SARC+/HF+	0.300
SPPB score	SARC-/HF-	SARC+/HF-	**<0.001**
	SARC-/HF-	SARC-/HF+	0.321
	SARC-/HF-	SARC+/HF+	**<0.001**
	SARC+/HF-	SARC-/HF+	0.916
	SARC+/HF-	SARC+/HF+	0.336
	SARC-/HF+	SARC+/HF+	0.482
FRIED score	SARC-/HF-	SARC+/HF-	**<0.001**
	SARC-/HF-	SARC-/HF+	0.992
	SARC-/HF-	SARC+/HF+	**<0.001**

	SARC+/HF-	SARC-/HF+	0.071
	SARC+/HF-	SARC+/HF+	0.315
	SARC-/HF+	SARC+/HF+	**0.012**
ADL score	SARC-/HF-	SARC+/HF-	**<0.001**
	SARC-/HF-	SARC-/HF+	1.000
	SARC-/HF-	SARC+/HF+	**<0.001**
	SARC+/HF-	SARC-/HF+	0.242
	SARC+/HF-	SARC+/HF+	0.120
	SARC-/HF+	SARC+/HF+	0.026
TUG (seconds)	SARC-/HF-	SARC+/HF-	**<0.001**
	SARC-/HF-	SARC-/HF+	0.146
	SARC-/HF-	SARC+/HF+	**<0.001**
	SARC+/HF-	SARC-/HF+	0.995
	SARC+/HF-	SARC+/HF+	0.990
	SARC-/HF+	SARC+/HF+	0.982
Hemoglobin (g/dL)	SARC-/HF-	SARC+/HF-	0.004
	SARC-/HF-	SARC-/HF+	0.898
	SARC-/HF-	SARC+/HF+	0.639
	SARC+/HF-	SARC-/HF+	0.287
	SARC+/HF-	SARC+/HF+	0.877
	SARC-/HF+	SARC+/HF+	0.604
Serum albumin (g/dL)	SARC-/HF-	SARC+/HF-	**<0.001**
	SARC-/HF-	SARC-/HF+	0.999
	SARC-/HF-	SARC+/HF+	**<0.001**
	SARC+/HF-	SARC-/HF+	0.309
	SARC+/HF-	SARC+/HF+	0.350
	SARC-/HF+	SARC+/HF+	0.078
HbA1c (%)	SARC-/HF-	SARC+/HF-	0.826
	SARC-/HF-	SARC-/HF+	0.695
	SARC-/HF-	SARC+/HF+	0.061
	SARC+/HF-	SARC-/HF+	0.536
	SARC+/HF-	SARC+/HF+	0.015
	SARC-/HF+	SARC+/HF+	0.997
Blood urea (mg/dL)	SARC-/HF-	SARC+/HF-	0.010
	SARC-/HF-	SARC-/HF+	0.937
	SARC-/HF-	SARC+/HF+	0.440
	SARC+/HF-	SARC-/HF+	0.389
	SARC+/HF-	SARC+/HF+	0.991
	SARC-/HF+	SARC+/HF+	0.568
Serum uric acid (mg/dL)	SARC-/HF-	SARC+/HF-	0.883
	SARC-/HF-	SARC-/HF+	0.861
	SARC-/HF-	SARC+/HF+	0.812
	SARC+/HF-	SARC-/HF+	0.946
	SARC+/HF-	SARC+/HF+	0.547
	SARC-/HF+	SARC+/HF+	0.644
CK (U/L)	SARC-/HF-	SARC+/HF-	0.133
	SARC-/HF-	SARC-/HF+	0.207

	SARC-/HF-	SARC+/HF+	0.178
	SARC+/HF-	SARC-/HF+	0.613
	SARC+/HF-	SARC+/HF+	0.857
	SARC-/HF+	SARC+/HF+	0.904
Serum vitamin D (ng/mL)	SARC-/HF-	SARC+/HF-	0.105
	SARC-/HF-	SARC-/HF+	0.887
	SARC-/HF-	SARC+/HF+	0.001
	SARC+/HF-	SARC-/HF+	0.460
	SARC+/HF-	SARC+/HF+	0.079
	SARC-/HF+	SARC+/HF+	0.056
NTproBNP (pg/mL)	SARC-/HF-	SARC+/HF-	0.231
	SARC-/HF-	SARC-/HF+	0.480
	SARC-/HF-	SARC+/HF+	**<0.001**
	SARC+/HF-	SARC-/HF+	0.867
	SARC+/HF-	SARC+/HF+	0.010
	SARC-/HF+	SARC+/HF+	0.826
IVS (mm)	SARC-/HF-	SARC+/HF-	0.037
	SARC-/HF-	SARC-/HF+	0.670
	SARC-/HF-	SARC+/HF+	1.000
	SARC+/HF-	SARC-/HF+	0.992
	SARC+/HF-	SARC+/HF+	0.363
	SARC-/HF+	SARC+/HF+	0.708
PWT (mm)	SARC-/HF-	SARC+/HF-	0.247
	SARC-/HF-	SARC-/HF+	0.144
	SARC-/HF-	SARC+/HF+	0.951
	SARC+/HF-	SARC-/HF+	0.408
	SARC+/HF-	SARC+/HF+	0.951
	SARC-/HF+	SARC+/HF+	0.337
LVEDD (mm)	SARC-/HF-	SARC+/HF-	0.768
	SARC-/HF-	SARC-/HF+	0.016
	SARC-/HF-	SARC+/HF+	0.001
	SARC+/HF-	SARC-/HF+	0.006
	SARC+/HF-	SARC+/HF+	**<0.001**
	SARC-/HF+	SARC+/HF+	0.863
LVESD (mm)	SARC-/HF-	SARC+/HF-	0.161
	SARC-/HF-	SARC-/HF+	0.012
	SARC-/HF-	SARC+/HF+	**<0.001**
	SARC+/HF-	SARC-/HF+	0.001
	SARC+/HF-	SARC+/HF+	**<0.001**
	SARC-/HF+	SARC+/HF+	0.996
LVEF (%)	SARC-/HF-	SARC+/HF-	0.863
	SARC-/HF-	SARC-/HF+	**<0.001**
	SARC-/HF-	SARC+/HF+	**<0.001**
	SARC+/HF-	SARC-/HF+	**<0.001**
	SARC+/HF-	SARC+/HF+	**<0.001**
	SARC-/HF+	SARC+/HF+	1.000

Abbreviations. n: number of patients; IADL: instrumental Activities of daily living; CIRS-G: Cumulative illness rating scale-geriatric version; CRP C-reactive protein; SARC-F: Strenght, assistance with walking rise from a chair, climb stairs and falls; HGS:

Handgrip strength; SPPB: Short Physical Performance Battery; ADL: activities of daily living; TUG: Timed Up and Go; HbA1c:: Glycated hemoglobin; CK: Creatine kinase; NT-proBNP: N-terminal pro-B-type natriuretic peptide; IVS: interventricular septal thickness; PWT: posterior wall thickness; LVEDD: left ventricular end-diastolic diameter; LVESD: left ventricular end-systolic diameter; LVEF: left ventricular ejection fraction .

Supplementary Table 2. Correlation between sarcopenia parameters and LVEF and ADL and FRIED scores among the patients' groups.

	all patients		SARC-/HF-		SARC+/HF-		SARC-/HF+		SARC+/HF+	
**Variable**	**R**	**p-value**	**R**	**p-value**	**R**	**p-value**	**R**	**p-value**	**R**	**p-value**
ADL score										
SPPB Score	-0.715	<0.001	-0.598	<0.001	-0.773	<0.001	-0.539	0.047	-0.691	<0.001
TUG falling	0.563	<0.001	0.517	<0.001	0.58	<0.001	0.378	0.182	0.421	0.007
LVEF	-0.165	<0.001	-0.195	0.003	-0.065	0.271	0.13	0.657	0.02	0.889
FRIED score										
SPPB Score	-0.703	<0.001	-0.713	<0.001	-0.634	<0.001	-0.784	0.001	-0.476	<0.001
TUG falling	0.603	<0.001	0.555	<0.001	0.582	<0.001	0.765	0.001	0.434	0.005
LVEF	-0.156	<0.001	-0.185	0.004	-0.048	0.41	0.644	0.013	-0.102	0.471

Abbreviation: ADL score: Activities of Daily Living score; SPPB score: Short Physical Performance Battery score; TUG falling: Fall risk based on Timed Up and Go test; LVEF:Left Ventricular Ejection Fraction; FRIED: Fried Frailty Phenotype score score.

Supplementary Table 3. Summary of the balance assessment.

Summary of Balance for All Data:							
	Means Treated	Means Control Std.	Mean Diff.	Var. Ratio	eCDF Mean	eCDF Max	
distance	0.11	0.05	0.81	1.90	0.24	0.48	
Age	78.31	81.65	-0.46	1.20	0.13	0.25	
Hb	13.24	12.33	0.34	1.69	0.15	0.38	
CK	56.57	82.44	-0.83	0.16	0.10	0.26	
Vitamin D	28.33	23.22	0.49	0.58	0.16	0.39	
Summary of Balance for Matched Data:							
	Means Treated	Means Control Std.	Mean Diff.	Var. Ratio	eCDF Mean	eCDF Max	Std. Pair Dist.
distance	0.11	0.10	0.15	1.16	0.02	0.25	0.22
age	78.31	78.90	-0.08	1.00	0.04	0.13	0.75
Hb	13.24	12.99	0.09	1.77	0.10	0.29	0.68
CK	56.57	53.79	0.09	0.98	0.05	0.23	1.09
Vitamin D	28.33	27.03	0.12	0.47	0.10	0.30	1.38

eCDF: empirical cumulative distribution function

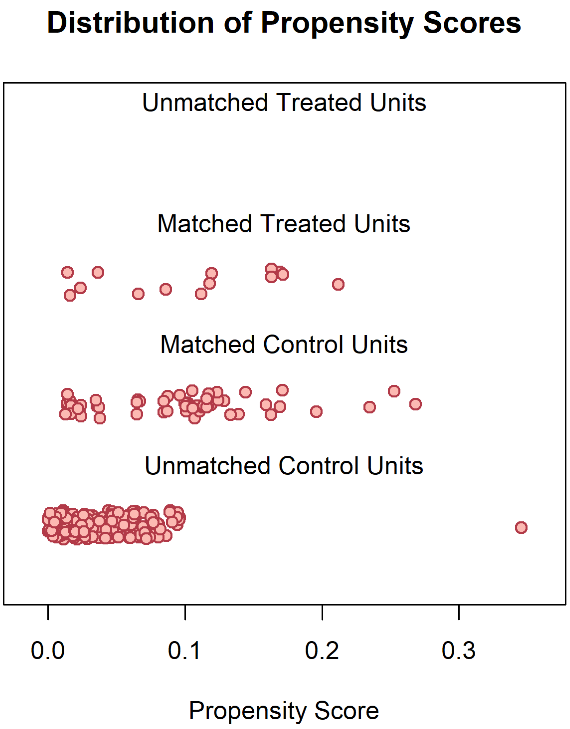

Supplementary Figure 1. Presentation of the propensity matching between patients from the SARC+/HF- group and those from the SARC-/HF+ group. A total of 56 SARC+/HF- patients were msthced with the 14 SARC-/HF+ cases.

Supplementary Table 4. Characteristics of the propensity matched patients.

Variable	SARC+/HF- n=56	SARC-/HF+ n=14	p-value
**Age, (years), median(q1;q3)**	77.42(73.36;84.13)	77.84(72.97;84.16)	0.789
**SPPB walk speed 4m (sec), median(q1;q3)**	0.33(0.14;0.8)	0.56(0.34;0.76)	0.242
**SPPB chair test (sec), median(q1;q3)**	27(17.14;60)	18.42(16.75;32.25)	0.401
**FRIED score, median(q1;q3)**	3(2;4)	2(1;3)	0.046
**ADL score, median(q1;q3)**	2.5(0;11)	0(0;0)	0.032
**TUG, median(q1;q3)**	18(13;22)	19(15.5;21.5)	0.973
**Hemoglobin (g/dL), median(q1;q3)**	13(11.97;14.43)	13.65(12.85;14.95)	0.697
**CK (U/L), median(q1;q3)**	45(31.25;72.25)	50(38.5;70.25)	0.766
**Serum Vitamin D (ng/mL), median(q1;q3)**	24.45(14.42;35.7)	27.56(22.8;35.39)	0.762

Abbreviation: SPPB: Short Physical Performance Battery; FRIED: Frailty Phenotype score score; ADL score: Activities of Daily Living score; TUG: Timed up and Go test; CK: Creatine kinase.
